# Molecular markers of anti-malarial drug resistance in Central, West and East African children with severe malaria

**DOI:** 10.1186/s12936-017-1868-y

**Published:** 2017-05-23

**Authors:** Christian N. Nguetse, Ayola Akim Adegnika, Tsiri Agbenyega, Bernhards R. Ogutu, Sanjeev Krishna, Peter G. Kremsner, Thirumalaisamy P. Velavan

**Affiliations:** 1 Institute of Tropical Medicine, University Tübingen, Wilhelmstrasse 27, 72074 Tübingen, Germany; 2 Fondation Congolaise pour la Recherche Médicale, Brazzaville, Republic of Congo; 3 Centre de Recherches Médicales de Lambaréné, Lambaréné, Gabon; 4 Department of Physiology, University of Science and Technology, School of Medical Sciences, Kumasi, Ghana; 5 Departments of Child Health and Medicine, Komfo Anokye Teaching Hospital, Kumasi, Ghana; 6 Centre for Clinical Research, Kenya Medical Research Institute, Kisumu, Kenya; 7 Institute for Infection and Immunity, St George’s University of London, London, UK; 8 Vietnamese-German Center for Medical Research, Hanoi, Vietnam; 9 Faculty of Medicine, Duy Tan University, Da Nang, Vietnam

**Keywords:** Malaria, *P. falciparum*, *Pfmdr1*, *Pfatp6*, *Pfk13*, Anti-malarial drugs, Resistance, Africa

## Abstract

**Background:**

The *Plasmodium falciparum* multidrug resistance 1 (PfMDR1), *P. falciparum* Ca^2+^-ATPase (PfATP6) and Kelch-13 propeller domain (PfK13) loci are molecular markers of parasite susceptibility to anti-malarial drugs. Their frequency distributions were determined in the isolates collected from children with severe malaria originating from three African countries.

**Methods:**

Samples from 287 children with severe malaria [(Gabon: n = 114); (Ghana: n = 89); (Kenya: n = 84)] were genotyped for *pfmdr1, pfatp6* and *pfk13* loci by DNA sequencing and assessing *pfmdr1* copy number variation (CNV) by real-time PCR.

**Results:**

*Pfmdr1*-N86Y mutation was detected in 48, 10 and 10% in Lambaréné, Kumasi and Kisumu, respectively. At codon 184, the prevalence of the mutation was 73% in Lambaréné, 63% in Kumasi and 49% Kisumu. The S1034C and N1042D variants were absent at all three sites, while the frequency of the D1246Y mutation was 1, 3 and 13% in Lambaréné, Kumasi and Kisumu, respectively. Isolates with two *pfmdr1* gene copy number predominantly harboured the N86Y wild-type allele and were mostly found in Kumasi (10%) (*P* < 0.0001). Among the main *pfmdr1* haplotypes (NFD, NYD and YFD), NYD was associated with highest parasitaemia (*P* = 0.04). At the *pfatp6* locus, H243Y and A623E mutations were observed at very low frequency at all three sites. The prevalence of the *pfatp6* E431K variant was 6, 18 and 17% in Lambaréné, Kumasi and Kisumu, respectively. The L263E and S769N mutations were absent in all isolates. The *pfk13* variants associated with artemisinin resistance in Southeast Asia were not observed. Eleven novel substitutions in the *pfk13* locus occurring at low frequency were observed.

**Conclusions:**

Artemisinins are still highly efficacious in large malaria-endemic regions though declining efficacy has occurred in Southeast Asia. The return of chloroquine-sensitive strains following the removal of drug pressure is observed. However, selection of wild-type alleles in the multidrug-resistance gene and the increased gene copy number is associated with reduced lumefantrine sensitivity. This study indicates a need to constantly monitor drug resistance to artemisinin in field isolates from malaria-endemic countries.

## Background

Malaria still claims hundreds of thousand deaths in sub-Saharan Africa, mostly children under the age of five and pregnant women. The spread of artemisinin-resistant *Plasmodium falciparum* parasites from Southeast Asia to Africa is foreseen. Following the current World Health Organization (WHO) recommendation, artemisinin-based combination therapy (ACT) is the first-line treatment of severe malaria [1]. Failure of ACT is well documented in Western Cambodia and the Thai-Myanmar border [2–4]. As a result, the WHO has developed strategies to contain the spread of resistant parasites to other countries [5].

It is clearly important to identify resistant phenotypes and constantly monitor for artemisinin resistance. Definitions of artemisinin resistance range from persistence of parasites on the third day after drug administration [6], an increased parasite clearance half-life [7, 8], a reduced parasite clearance rate [3, 9, 10] and, finally, a treatment failure of ACT [11], although these are debated [11–14] and ACT failures also arise because of failure of partner drugs of artemisinins. The *P. falciparum* multidrug resistance 1 (PfMDR1) locus on chromosome 5 and the *P. falciparum* Ca^2+^-ATPase (PfATP6) locus on chromosome 1 genes may also modulate susceptibility of the parasites to artemisinins [15–18]. PfATP6, a candidate marker for artemisinin susceptibility [16, 19], is a sarco-endoplasmic reticulum calcium-ATPase (serca)-type calcium pump of the parasite suggested to be the target for artemisinins [15]. Increase in the gene copy number (copy number variation, CNV) of *pfmdr1* [20–22], which encodes an ATP-binding cassette transporter homologue of the P-glycoprotein 1 [23] is also involved in multidrug resistance, including to artemisinins [24]. Recently, a new locus was identified, the kelch protein located on chromosome 13 (PfK13 propeller); *pfk13* mutations associated with clinical artemisinin resistance in Cambodia [25], and in vitro ring-stage survival rates [26], have now spread across Southeast Asia and South China [27, 28] and have shown to also arise independently [29]. For these reasons, mutations in the *pfatp6*, *pfmdr1* and *pfk13* genes may be useful molecular markers of artemisinin resistance [25, 30–32].

This is a sub-study conducted by the “Severe Malaria in African Children” (SMAC) consortium [33]. The goal of this study was to describe parasite polymorphisms at the *pfmdr1*, *pfatp6* and *pfk13* loci, including the *pfmdr1* copy number in West, Central and East African children with severe malaria.

## Methods

### Study design and sample collection

Two hundred and ninety-six children aged 0.5–10 years (120 in Lambaréné, Gabon, 90 in Kumasi, Ghana, 86 in Kisumu, Kenya) were randomly selected from the SMAC study [33]. Blood samples from all patients were collected in heparinized tubes. Storage and transport of specimens were done using a cold chain transport tool for subsequent molecular analyses.

### PfMDR1, PfATP6 and PfK13 genotyping

Genomic DNA was isolated using the QIAamp DNA mini blood kit (Qiagen, Hilden, Germany). The *pfmdr1* mutations N86Y, Y184F, S1034C, N1042D and D1246Y were screened by nested PCR using primer pairs described elsewhere [34]. The *pfatp6* mutations H243Y, L263E, E431K, A623E and S769N were screened by PCR using the primer pairs designed by Zakeri et al. [35]. Mutations M476I, Y493H, R539T, I543T and C580Y in the *pfk13* gene were screened using the primer pairs indicated by Ariey et al. [25]. In brief, 10 ng of parasite genomic DNA were added to a 20 µL reaction mixture containing 1× PCR buffer (20 mM Tris–HCl pH 8.4, 50 mM KCl, 2.5 mM of MgCl_2_), 0.125 mM of dNTPs, 0.25 mM of each primer and 1U Taq DNA polymerase (Qiagen, Hilden, Germany). The PCR reaction was run on a PTC-200 Thermal cycler (MJ Research, Waltham, USA). PCR products were visualized through electrophoresis on a 1.2% agarose gel stained with SYBR green I in 1× Tris-electrophoresis buffer (90 mM Tris–acetate, pH 8.0, 90 mM boric acid, 2.5 mM EDTA). Subsequently, PCR products were purified (Exo-SAP-IT, USB, Affymetrix, Santa Clara, CA, USA) and directly used as templates for DNA sequencing using the BigDye terminator v. 1.1 cycle sequencing kit (Applied Biosystems, Foster City, USA) on an ABI 3130XL DNA sequencer. Polymorphisms were identified by assembling the sequences with the reference sequence of the *pfmdr1* (NC_004326.1), *pfatp6* (NC_004325.1) and *pfk13* (NC_004331.2) genes using the Codoncode Aligner 4.0 software and visually reconfirmed from their electropherograms.

### PfMDR1 copy number

The *pfmdr1* gene copy number was estimated by TaqMan real-time PCR using the hydrolysis probes as previously described [36]. In brief, 10 ng of genomic DNA were added to a 25 µL reaction mixture containing 1× TaqMan buffer [8% glycerol, 0.625U DNA polymerase, 5.5 mmol/L MgCl_2_, 300 µmol/L dNTPs, 600 nmol/L passive reference dye ROX (5-carboxy-X-rhodamine), pH 8.3], 300 nmol/L of each primer, 100 nmol/L of each probe, and 5 µL of the template DNA. The real-time PCR reaction was run on a Corbett device (Research RG-3000, Qiagen, Hilden, Germany). The thermal conditions after a pre-incubation step (95 ^°^C, 5 min) were 50 cycles of 95 ^°^C for 15 s and 58 ^°^C for 1 min. Genomic DNA from *P. falciparum* reference strain 3D7 was used as calibrator and *P. falciparum* ß-tubulin, a house-keeping gene, was used as reference gene. For multiple *pfmdr1* gene copy numbers, DNA from the Dd2 clone was used as control. The 2^−ΔΔCt^ method of relative quantification was used to estimate the gene copy number [37]. Each sample was run in triplicate along with the reference DNA samples from clones 3D7 and Dd2, which are known to have a *pfmdr1* gene copy number of 1 and 2–4, respectively. The mean and standard deviation of the three threshold cycle (Ct) values were calculated for each sample. The experiment was repeated if one of the following results was obtained: ΔΔCt spread >1.5; Ct values >35; or copy number value = 1.3–1.6 [36]. Copy number estimates were rounded to the nearest integer and parasites with more than 1.5 copies were considered multiple copies [36]. A sample was considered to carry one copy of the gene if the N-fold copy number was between 0.5 and 1.5 (0.5 < N-fold < 1.5).

### Statistical analysis

Data were analysed using GraphPad Prism (GraphPad software Inc, La Jolla, CA). Mann–Whitney U and Kruskal–Wallis H One-way ANOVA tests were executed to determine possible associations of parasitaemia with the increased gene copy number and with the haplotypes of the genes investigated. Dunn’s multiple comparison test was used to test for differences among study sites. The level of significance was set to a *P* value of <0.05.

## Results

In total, 296 children with severe malaria were enrolled in this study. Available for genetic analyses were 287 patients.

### PfMDR1 polymorphisms

All 287 samples were successfully genotyped for *pfmdr1* mutations. The frequency distribution of genetic variants identified is given in Table 1. The S1034C and N1042D mutations were absent at all three sites, while the frequency of the D1246Y mutation was 1, 3 and 13% in Lambaréné, Kumasi and Kisumu, respectively.

**Table 1:** Frequency distribution of mutant *pfmdr1* and *pfatp6* alleles in isolates from Lambaréné, Kumasi and Kisumu

Site (N)	Number (%) of samples with allelic change at these codons					
	*Pfmdr1*			*Pfatp6*		
	86Y	184F	1246Y	243Y	431K	623E
Lambaréné (114)	55 (48)	83 (73)	1 (1)	1 (1)	6 (6)	1 (1)
Kumasi (89)	9 (10)	56 (63)	3 (3)	1 (1)	13 (18)	1 (1)
Kisumu (84)	8 (10)	41 (49)	11 (13)	2 (3)	13 (17)	1 (1)

There were no mutant alleles at the following codons: *pfmdr1* S1034C, N1042D and *pfatp6* L263E, S769N

In Lambaréné, the prevalence of the wild-type N86Y allele was 40%. The frequency of mixed genotypes was 12%. At codon 184, the frequency of the wild-type and mutant Y184F alleles was 25 and 73%, respectively, whereas the prevalence of mixed genotypes was 2%. The double mutations N86Y/Y184F, and N86Y/D1246Y were observed in 51 (45%) and 1 (1%) samples, respectively.

In Kumasi, the majority of samples (73%) carried the wild-type N86Y allele. The prevalence of mixed genotypes was 17%. The prevalence of the wild-type Y184F allele was 30, and 7% for the mixed genotypes. The prevalence of double mutations N86Y/Y184F, and N86Y/D1246Y was 9 and 1%, respectively. There was only one sample with the triple mutant N86Y-Y184F-D1246Y allele.

In Kisumu, 82% of samples carried the wild-type allele N86Y, and 8% had mixed genotypes. The frequency of samples carrying the wild-type Y184F allele 44%. The samples with mixed genotypes were 7%. Two samples (2%) had the double mutant N86Y/Y184F, and 4 (5%) had the double mutant N86Y/D1246Y. One sample carried the triple mutant N86Y-Y184F-D1246Y allele.

### PfMDR1 copy number

**Fig. 1:**
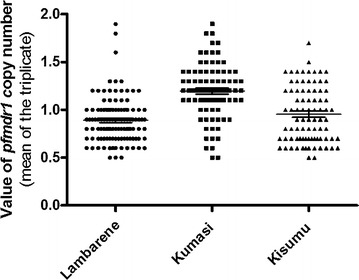
Distribution of *pfmdr1* gene copy number in isolates from Lambaréné, Kumasi and Kisumu

The copy number of the *pfmdr1* was successfully determined and results were available for 285 isolates. Two isolates, one each in Lambaréné and Kisumu, were removed from the analysis because of inconsistent results. The overall *pfmdr1* copy number mean was 0.89 (range 0.5–1.9), 1.2 (range 0.5–1.9) and 0.95 (range 0.5–1.7) in Lambaréné, Kumasi and Kisumu, respectively. With the control DNA, the results were reproducible with a mean copy number of 1.1 and a standard deviation of 0.18 for the 3D7 strain. The Dd2 control gave a mean copy number of 3.98 with a standard deviation of 0.38. When rounded to an integer, three, nine and one isolates with two gene copy numbers were observed in Lambaréné, Kumasi and Kisumu, respectively. The values of the *pfmdr1 copy* number from the three study sites are depicted in Fig. 1. When rounded to the nearest integer, 1 gene copy number was found in 97, 90 and 99% isolates from Lambaréné, Kumasi and Kisumu, respectively. There was a significant difference of the *pfmdr1* copy number among the parasites from the three study sites (*P* < 0.0001). Dunn’s multiple comparison showed that parasites retrieved in samples from Kumasi had more copy numbers than identified in those from Lambaréné and Kisumu. In this study, the *pfmdr1* copy number was not related to the baseline parasitaemia [Mean parasitaemia, Kisumu = 183,233 (range 7452–1,677,780), Kumasi = 171,835 (range 6240–1,209,000) and Lambaréné = 80,986 (range 5376–745,100)]. The isolates with an increased copy number were found to harbour predominantly the N86Y wild-type allele alone (9/13) or combined with the mutant allele (11/13) compared to the mutant allele N86Y alone (2/13).

### PfMDR1 haplotypes

The *pfmdr1* haplotypes were reconstructed including the mutations N86Y, Y184F and D1246Y. The isolates with a mixture of two haplotypes were each counted as carrying both. In total, eight different haplotypes were observed. In Lambaréné, the haplotype YFD was the most prevalent one, occurring at a frequency of 57%, followed by NFD (30%), NYD (24%), YYD (5%) and YYY (1%). In Kumasi, the most prevalent haplotype was NFD (60%) followed by NYD (34%), YFD (23%), YYY (3%) and NYY (2%). The other haplotypes NFY, YYD and YFY all occurred at a frequency of 1%. In Kisumu, the haplotype NFD was the most prevalent, occurring at a frequency of 51%, followed by NYD (37%), NYY and YFD both occurring at a frequency of 7%, YYD and YYY each at 6%, NFY (2%) and YFY (1%). The most prevalent haplotypes associated with increased *pfmdr1* copy number were NFD, NYD and YFD occurring six, five and four times, respectively. For these main haplotypes (NFD, NYD and YFD), the mean parasitaemia was significantly different (*P* = 0.04) and this difference was observed between the haplotypes NYD and YFD (*P* = 0.012).

### PfATP6 polymorphisms

Of the 287 collected samples, only 250 samples (99/114, 74/89 and 77/84 in Lambaréné, Kumasi and Kisumu, respectively) were successfully genotyped for *pfatp6* mutations (Table 1). In the samples collected in Lambaréné, the prevalence of the *pfatp6* H243Y, E431K and A623E mutant alleles was 1, 6 and 1%, respectively. The frequency of the mixed genotypes at codon 243 was 1%. In Kumasi, two isolates harboured the mixed genotypes at codons 243 and 623 at a frequency of 1% each, respectively. Thirteen (18%) samples carried the mutant allele E431K while two samples (1%), one each carried the mutant H243Y and A623E alleles, respectively. In Kisumu, the frequency of the mutant alleles E431K, H243Y and A623E was 17, 3 and 1%, respectively. The prevalence of the mixed genotypes only found at codon A623E was 3%.

### PfATP6 haplotypes

The *pfatp6* haplotypes were reconstructed using the mutations H243Y, E431K and A623E. Isolates with a mixture of two haplotypes were counted as carrying both. Five haplotypes were investigated. The HEA haplotype was the most prevalent one, occurring at a frequency of 92, 80, and 81% in Lambaréné, Kumasi and Kisumu, respectively. In Lambaréné, the other haplotypes were HKA, HEE and YEA occurring at frequencies of 6, 1 and 2%, respectively. In Kumasi, the haplotypes HKA, HEE, YEA and YKA were found at frequencies of 18, 3, 1 and 1%, respectively. In Kisumu, the haplotypes HKA, HEE and YEA were found at frequencies of 17, 4 and 3%, respectively. There was no association of the mean parasitaemia with any of the haplotypes.

### PfK13 polymorphisms

The nonsynonymous mutations M476I, Y493H, R539T, I543T and C580Y, which were previously shown to be associated with an increased parasite clearance half-life time in Cambodia were absent in these samples. However, several yet unrecognized variants were identified individually in eleven parasites (three, two and six in Lambaréné, Kumasi and Kisumu, respectively), albeit at very low frequencies (Table 2).

**Table 2:** Mutations in the Kelch-13 propeller domain (*pfk13*) of isolates from Lambaréné, Kumasi and Kisumu

Codon position	Amino acid reference	Nucleotide reference	Amino acid mutation	Nucleotide mutation	Site
549	S	tc**t**	S	tc**C**	Kumasi
553	P	cc**g**	P	cc**A**	Kisumu
578	A	**g**ct	S	**T**ct	Kisumu
589	V	**g**tc	I	**A**tc	Lambaréné
589	V	gt**c**	V	gt**T**	Lambaréné
639	G	gg**t**	G	gg**C**	Kumasi
645	N	aa**c**	N	aa**T**	Lambaréné
666	V	gt**a**	V	gt**G**	Kisumu

## Discussion

In the absence of an effective malaria vaccine, treatment failures associated with ACT (TFACT) will hamper global efforts in reducing malaria mortality and morbidity. Besides studies on Africans, one of the most affected populations, highlighted the significant contribution of host genetics towards the stabilisation of these two indicators of health status [38–41]. A previous study has shown for the first time that the *pfmdr1* N86Y polymorphism is associated with delayed parasite clearance when artesunate was used in monotherapy [33]. This finding may be of importance, as it demonstrates in vivo modulation of the efficacy of artesunate, the drug of choice against severe malaria. However, there is an important need to clearly define artemisinin resistance owing to the large number of definitions found in the literature. Besides inclusive debates are rather welcome if the goal is to tackle the problem of multidrug-resistant parasites [12]. In the meantime, continuous monitoring of the molecular markers of anti-malarial drug resistance will help to prevent the spread of resistant strains and serve as basis for future update of the treatment policy.

Concerning the frequency distribution of the *pfmdr1* mutations, when comparing with previous studies [42], an increased prevalence of the wild-type allele N86Y was observed in Gabon. This allele is reported to be associated with exposure to the anti-malarial drug combination artemether–lumefantrine (AL) [43, 44]. The mutation N86Y has previously been associated with a lower 50% inhibitory concentration (IC50) for artemisinin and dihydroartemisinin, as compared with the wild-type allele [18]. However, a recent study showed that the *pfmdr1* N86Y variant was associated with prolonged parasite clearance in vivo [33]. Its high frequency in Lambaréné might be alarming but parasites in that region are still sensitive to artemisinins. Further studies assessing the sensitivity of these parasites over time are needed. In Kumasi, the findings reported here were consistent with the study published by Kwansa-Bentum et al. [45]. In Kisumu, the prevalence of the *pfmdr1* wild-type allele N86Y was higher compared to a previous study [46]. This allele has been associated in vitro with a three to fourfold increase of the lumefantrine IC50 values, as compared to the mutant N86Y allele [47], and suggested together with other *pfmdr1* polymorphisms to be important determinants of parasite sensitivity to anti-malarials [48]. In addition, the *pfmdr1* wild-type allele N86Y is reported to be predominant in recurrent infections [49] and to increase in a fivefold degree the risk of recrudescence in individuals treated with AL [50]. These findings support the need of a constant monitoring of the parasite susceptibility to AL in Kumasi and Kisumu.

The mutations S1034C and N1042C, which have been reported to reduce parasite resistance to mefloquine [51] were not observed at all in this study. Regarding the variant D1246Y, a threefold increased prevalence from 4% [46] to 13% in Kisumu, compared to the lower prevalence of D1246Y of 9% [42] to 1% in Lambaréné, and of 14% [52] to 3% in Kumasi was noted. Sidhu et al. [53] have reported that the combination of the mutant alleles S1034C-N1042D-D1246Y, which occurs frequently in South America, was associated with increased parasite susceptibility to artemisinin. However, in this study, S1034C and N1042C were absent, except D1246Y. This finding suggests that there is some selective pressure exerted by the parasite on the drug transporter *pfmdr1* gene at codons 1034 and 1042. The selection of the wild-type alleles following ACT use is worrying as a higher virulence of these wild-type genotypes has been linked to febrile illness [54].

PfMDR1 gene amplification has been reported to be selected by the use of mefloquine, artesunate, lumefantrine and quinine [36, 52, 55–57]. Uhlemann et al. [56] found 5% of *P. falciparum* isolates in Lambaréné carrying two copies of the *pfmdr1* gene, while 7 years later the same authors did not identify any isolates with multicopies. In the present study, 3% of the isolates were found carrying two copies. This indicates a possible circulation of parasites carrying several copies of the *pfmdr1* gene in Lambaréné at a low prevalence. The low frequency of isolates with *pfmdr1* multicopies observed in Kisumu is consistent with previous studies [58, 59]; and in Ghana, these findings are in agreement with previous work [52, 60]. The *pfmdr1* gene amplification is one of the best indicators for monitoring parasite resistance to treatment with artesunate mefloquine combination, and some but not all parasites with increased *pfmdr1* gene copies had a reduced sensitivity to artesunate in the Thai-Myanmar border region [61]. Thus, the determination of *pfmdr1* gene amplification might become an appropriate tool for the assessment of parasite susceptibility in Ghana [52]. The majority of isolates with two copies of the *pfmdr1* gene also carried the wild-type N86Y allele compared to a few only harbouring the mutant allele. This suggests that the wild-type allele has a selective advantage to be amplified, although the mutant allele can also have its gene amplified [58].

The PfMDR1 haplotype YFD has been reported to be associated with increased parasite susceptibility to artemisinin derivatives, mefloquine, halofantrine and lumefantrine [22, 62, 63]. The high frequency of this haplotype in Lambaréné suggests that it contributes to maintain parasite susceptibility to artemisinin derivatives, compared to Kumasi and Kisumu where the haplotype was found at low frequencies. The *pfmdr1* NFD haplotype identified at higher prevalences in Kumasi and Kisumu than in Lambaréné might decrease parasite susceptibility to AL as it appears to be selected by AL [43, 64, 65]. Therefore, it must be well monitored in countries using AL [52], as the wild-type allele N86Y and potentially its duplication are associated with decreased parasite susceptibility to AL [36, 44]. Moreover, Malmberg et al. [49] showed that NFD followed by the other NYD, YYY and YYD haplotypes was significantly linked with resistance to higher lumefantrine concentration in vivo in Tanzania.

The *pfatp6* L263E and S769N mutations have been shown to reduce by allelic exchange parasite susceptibility to some artemisinins [16, 66] and to increase the IC50 of artemether [19]. These variants were not observed in these samples. L263E was not present in these isolates compared to the Greater Mekong subregion [67] though identified in field isolates from Tanzania after ACT were introduced [68]. Described as a potential molecular marker for artemisinin resistance [19], the S769N mutation was not present in this study population. Numerous studies have reported this mutation to be rare or absent [67, 69–75]. However, when assessed in African isolates, this mutation did not correlate with an increased IC50 to dihydroartemisinin, the main active compound of artemisinin derivatives [76] as investigated with the increased IC50 of artemether in isolates from French Guiana [19, 69, 77]. An explanation could be that either the functionality of this mutation is only linked to artemether, or that the distinct genetic makeup of the isolate plays a role [76], or that the frequency is still too low to allow for reliable conclusions. H243Y was found with low frequencies in the present study. However, its role in modulating artemisinin resistance is not well characterized [76]. The polymorphism E431K, reported by Jambou et al. to be linked with an increased artemether IC50 [19], was the most prevalent in this study. A previous study has reported an increased frequency of the A623E mutation after the implementation of ACT in Niger [75], while its prevalence in this study was low.

The *pfk13* is a polymorphic gene located 5.9 kb upstream of the 35 kb region. It is linked with delayed parasite clearance [78] and within the region of top-ranked signatures of selection on chromosome 13 [79]. Therefore, it has been proposed to be a reliable marker for artemisinin resistance [25]. Among the polymorphisms investigated by Ariey et al. [25] the M476I variant was acquired in vitro in the F32-ART5 *P. falciparum* lineage following increased concentrations of artemisinin. This polymorphism was absent in these isolates, suggesting that the development of artemisinin resistance in the field might differ in vivo and in vitro. The polymorphisms Y493H, R539T and C580Y, associated with a significant slow-clearing of parasites were also absent in this study. Even E612D found in Gambia [80] was absent in these isolates, again indicating the enormous heterogeneity of malaria parasites in Africa. The absence of these *pfk13* mutations in African isolates highlights two facts: (1) the mechanisms of artemisinin resistance in Africa are not identical with those observed in Southeast Asia and (2) artemisinin resistance occurring in Cambodia and Thailand has not spread to Africa yet.

Artemisinin and its derivatives remain highly efficacious in the treatment of malaria, including in Africa. The declining efficacy of ACT (TFACT) reported in Southeast Asia threatens the global efforts towards the elimination and eradication of the disease. Selective pressure on the multidrug-resistance gene favoured the return of chloroquine-sensitive (wild-type *pfmdr1* N86Y allele) strains as observed in this study and supported by accumulated evidences from previous studies [81, 82]. However, these alleles have been associated with decreased lumefantrine sensitivity. Furthermore, a re-emergence and persistence of *P. falciparum* isolates with multicopies of the *pfmdr1* gene in some parts of Africa were noted. This study indicates the need to continuously monitor parasite susceptibility to artemisinin and its derivatives in field isolates from malaria-endemic countries.

## Abbreviations

*PfMDR1*: *Plasmodium falciparum* multidrug resistance 1
*PfATP6*: *Plasmodium falciparum* Ca^2+^-ATPase
*PfK13*: *Plasmodium falciparum* Kelch-13 propeller domain
CNV: copy number variation
ACT: artemisinin-based combination therapy
IC50: half maximum inhibitory concentration
SMAC: Severe Malaria in African children
WHO: World Health Organization
